# Supplementary figures and images for: Accumulation of di-2-ethylhexyl phthalate from polyvinyl chloride flooring into settled house dust and the effect on the bacterial community
Source: PeerJ. 2019 Nov 22;7:e8147. doi: 10.7717/peerj.8147 (PMC6876486; doi:10.7717/peerj.8147)

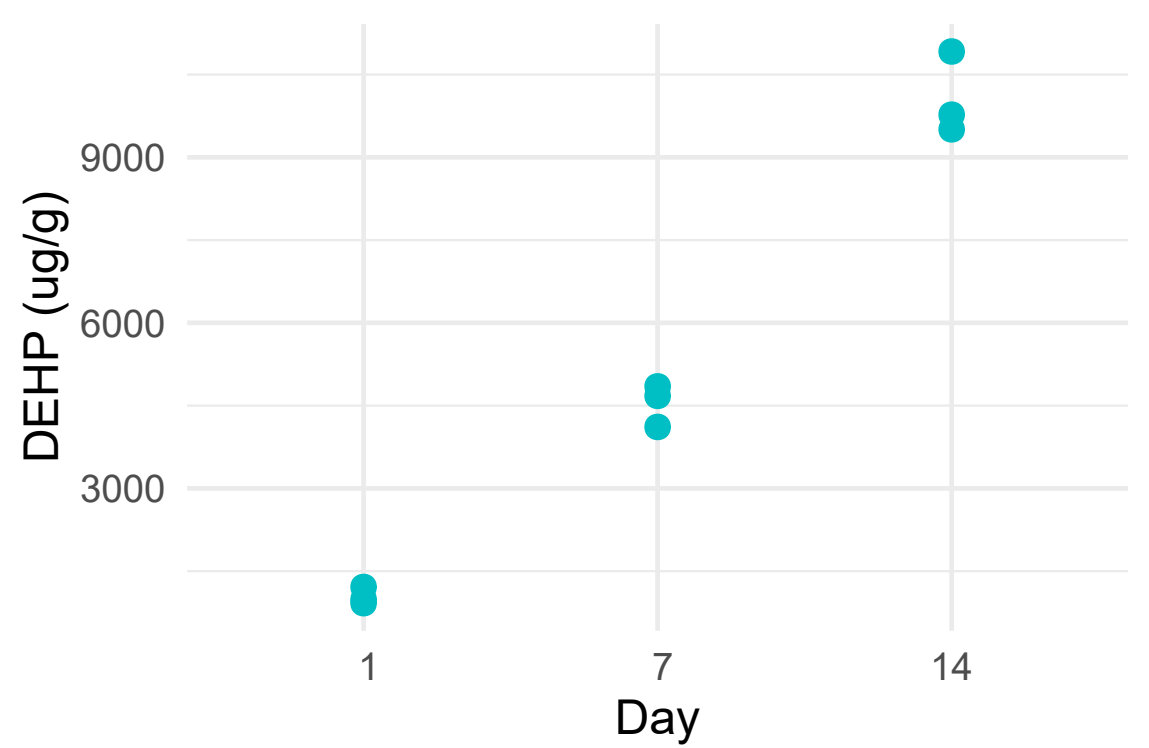

Supplement: Supplemental Information 1 [file peerj-07-8147-s001.pdf]

Sample type ● Control ● Experimental

**A**

Observed Richness (ASVs)

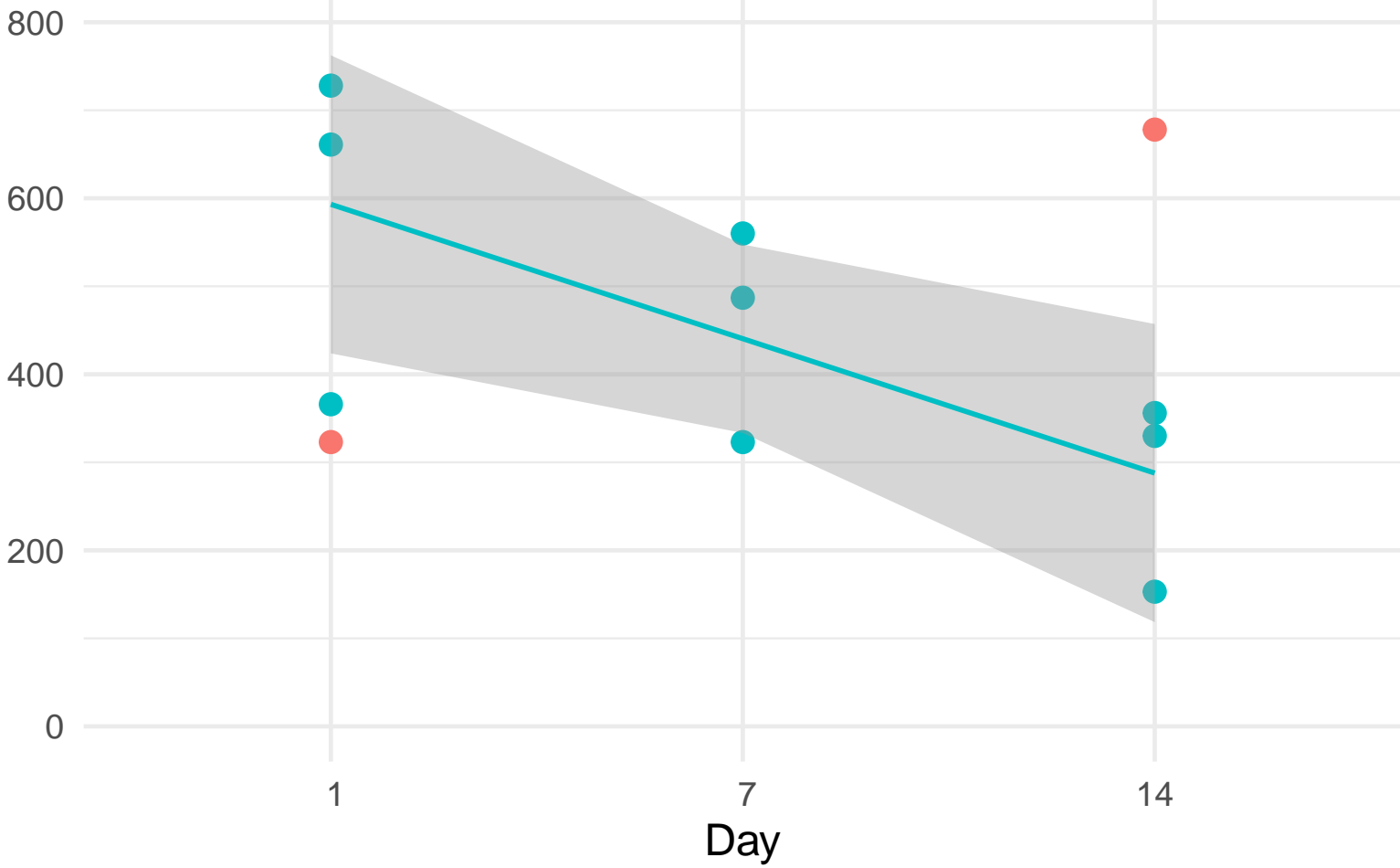

**B**

Shannon Diversity

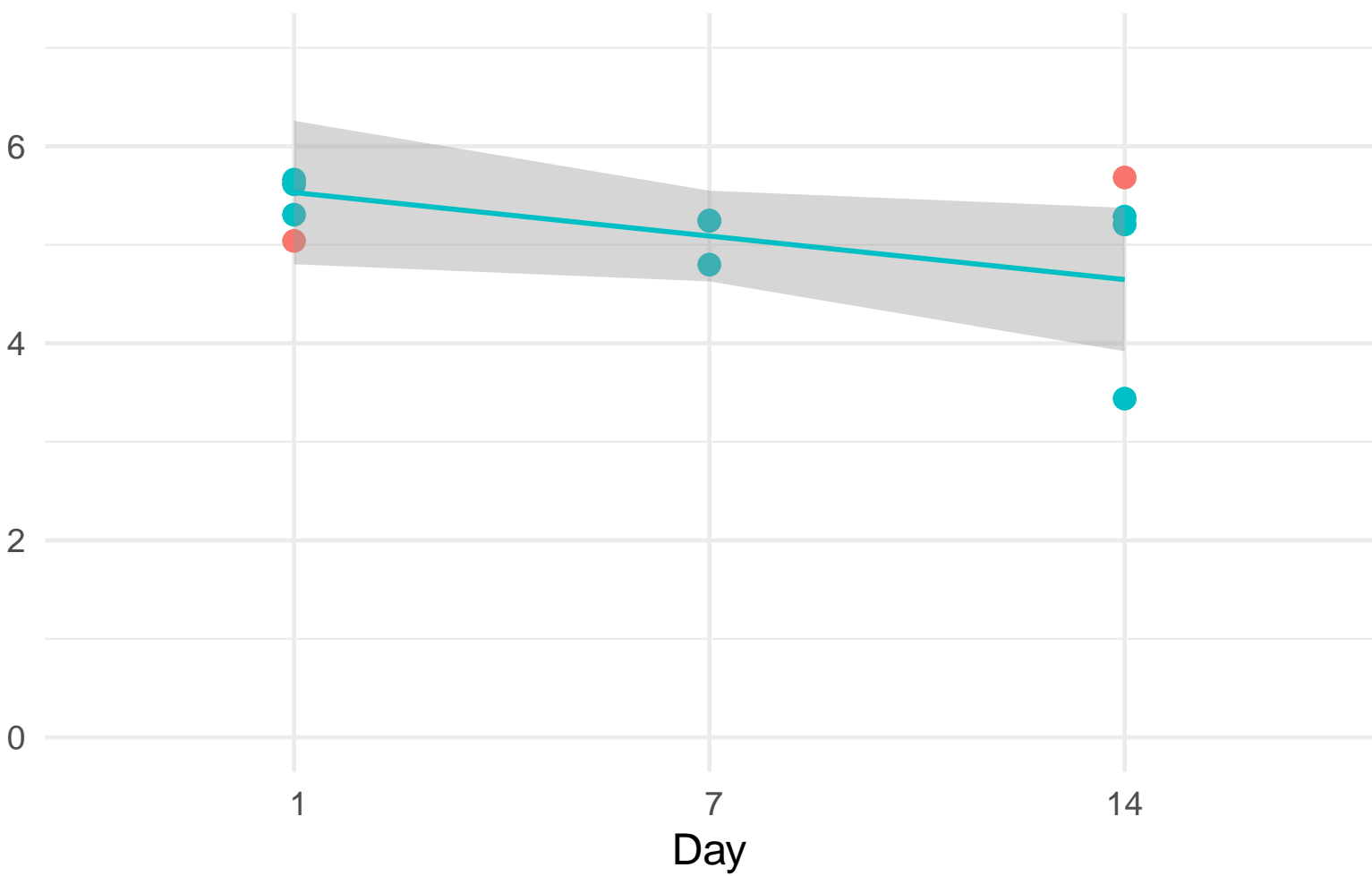

Supplement: Supplemental Information 2 [file peerj-07-8147-s002.pdf]

Control

Experimental

Relative Abundance

6000

4000

2000

0

1

1

1

1

1

7

7

7

14

14

14

Day

## Phylum

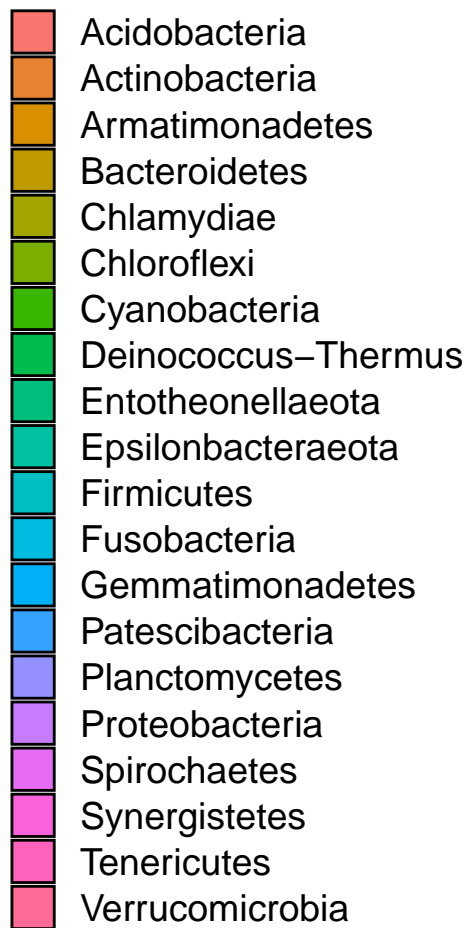

Supplement: Supplemental Information 3 [file peerj-07-8147-s003.pdf]
